# Role of Nanobubble Cavitation in Triggering Drug Release from Boron-Nitride and Carbon Nanocapsules and Their Diffusion for Drug Delivery Applications: A Molecular Dynamics Study

**DOI:** 10.3390/ijms26199582

**Published:** 2025-10-01

**Authors:** Farshad Heydarian, Sahar Rajabi Moghadam, Maryam Ghasemi, Elham Saniei, Sasan Rezaee, Ebrahim Kadivar, Ould el Moctar

**Affiliations:** 1 Department of Medical Radiation Engineering, CT. C., Islamic Azad University, Tehran 1477893855, Iran; 2 Institute of Sustainable and Autonomous Maritime Systems, University of Duisburg-Essen, 47057 Duisburg, Germany; sasan.rezaee@uni-due.de (S.R.); ould.el-moctar@uni-due.de (O.e.M.)

**Keywords:** drug delivery, diffusivity, nanobubble cavitation, carbon nanocapsules (CNs), boron nitride nanocapsules (BNNs)

## Abstract

Drug delivery is a well-established method for transporting anticancer drugs to cancerous tumors while minimizing damage to surrounding healthy tissues. Carbon nanocapsules (CNs) and boron nitride nanocapsules (BNNs) are promising nanocarriers capable of delivering drugs to tumor sites following their release. In this context, their diffusivity characteristics and drug release behavior need to be thoroughly addressed. This study examines the diffusion mechanisms of CNs and BNNs, as well as the impact of nanobubble cavitation on their performance as drug-releasing agents, utilizing molecular dynamics (MD) simulation methods. The results revealed that BNNs exhibit a higher diffusion coefficient compared to CNs in pure water. Moreover, temperature cannot be employed as a navigation mechanism for either CNs or BNNs. In terms of drug release, the collapse of nanobubbles at 298 K and 1 atm generates a high-energy water nanohammer, characterized by a temperature of approximately 1000 K and a pressure of 25 GPa, which impacts the nanocapsules. The impulse from the water nanohammer crushes the CN nanocapsule, whereas it leads to wall breakage in the BNN nanocapsule. Although both crushing and breakage can enable drug release, the crushing of CNs presents a higher risk of damage to the encapsulated drug. In summary, BNNs demonstrate better diffusivity and more favorable drug release behavior under nanobubble cavitation. However, further investigation is required to address targeting mechanisms and safer release strategies, involving the use of metallic functional groups and beam radiation, respectively.

## 1. Introduction

Cancer is one of the most critical health issues, claiming numerous lives worldwide regardless of gender or age [1,2]. Chemotherapy and radiation therapy, as two of the most potent strategies, are employed to mitigate, stop, and eliminate cancerous tumors [3]. In both methods, anticancer drugs must act on the cancerous tumors and destroy them. To achieve this goal, drugs such as Cisplatin [4] must be carried by drug carriers to reach the cancerous tumors. Near the cancerous tumors, the walls of the drug carriers break down under endogenous stimuli (e.g., changes in pH or redox gradients) or exogenous stimuli (e.g., temperature, magnetic fields, sound, or light waves), resulting in the release of the drug [5,6]. Consequently, the drug acts on cancerous tumors, damaging and deactivating them. This strategy ensures that the active anticancer drug is delivered directly to the tumors while minimizing its interaction with healthy tissues. As a result, the treatment’s effectiveness increases, and the response time is reduced. In these methods, both the drug carriers and the drug release mechanisms play critical roles and must be carefully chosen for each purpose.

Drug carriers are biocompatible cages that encapsulate anticancer drugs, keeping them safe and preventing contact with healthy tissues from the point of injection until the release stage [7,8,9,10]. They are typically designed using nanoparticles, nanotubes, nanocapsules, dendrimers, liposomes, hydrogels, or natural drug carriers [11,12,13,14,15]. The drug release stage involves the controlled release of drugs and their delivery to cancerous tumors under endogenous or exogenous stimuli [16,17,18]. Both of these components need to be accurately understood and carefully selected for use in drug delivery systems. In recent decades, significant progress in this field has been achieved through global research efforts [19,20,21,22,23,24,25,26]. As a state-of-the-art result, nanocapsules have been reported as efficient nanocarriers [22,23,24,25,26], and bubble cavitation [19,20,21] has been identified as an effective method for their release.

Nanocapsules, due to their small scale and self-assembly characteristics, can be promising candidates for drug carrier applications. Previous studies on these aspects have revealed that for two-end-open nanocapsules, commonly known as nanotubes, the releasing agent can be designed based on iron and gold nanowires [4,23]. Doping the wall of drug carriers affects the drug-releasing process [4]. The well-known materials used for carriers include carbon, silicon-carbide, and boron-nitride [4,22,23,25,27]. The anticancer drugs that these carriers can carry include temozolomide and carmustine for silicon-carbide [22], cisplatin [22], gemcitabine [23], and antimicrobial peptides [25] for carbon materials, as well as all of the above for boron-nitride [28,29,30,31,32]. Moreover, the self-assembly of nanocapsules in pure water facilitates the encapsulation of anticancer drugs within their carriers, demonstrating the practical applicability of nanocapsules [24]. Although these studies provide in-depth knowledge regarding drug carriers, the diffusion of these carriers, which signifies their mobility in locating and reaching cancerous tumors from the injection to delivery stages in the human body, remains almost unclear and needs to be addressed.

In the case of cavitation bubbles used during the drug release stage, the literature [19,20,21,33,34] reveals that the emission of acoustic waves near cancerous tumors, induces nano- and micro-bubbles by changing the pressure of the liquid surrounding the tumors through expansion and contraction cycles. These induced bubbles collapse, creating jets directed toward the carriers or the cancerous tumors. The impact of the jet on the carriers leads to their rupture and the release of anticancer drugs. In contrast, the direct effect of the jet on the cancerous tumors causes damage, and over prolonged treatment times, it eventually leads to deactivation. However, the mechanisms of jet formation and its effects on the carriers remain unclear and require further investigation.

To address these two main aspects (diffusion phenomena and drug release by nanobubble cavitation), the current study focuses on the diffusivity behavior of carbon and boron-nitride nanocapsules in pure water in the first section. Subsequently, the effects of nanobubble cavitation on nanocapsules will be investigated to provide a clearer insight into the interaction between drug carriers and the nanojets formed after the collapse of nanobubbles. All analyses will be conducted using the molecular dynamics (MD) method, a powerful technique for drug delivery research [22,23,24,25,26]. Carbon and boron-nitride nanocapsules were chosen due to their applicability in carrying a wide range of anticancer drugs. At the same time, water was chosen as the medium because the human body is primarily composed of water. The results obtained from the first section will aid in selecting the optimal nanocapsule material for enhanced migration and drug-carrying efficiency. The results from the second section will elucidate the interaction of nanocapsules with nanojets at the atomic scale. the novelty of this paper lies in two areas, the diffusion behavior and the effects of cavitation-induced damage on carbon nanocapsules and boron nitride nanocapsules, which can provide a deeper understanding of the underlying physics to advance drug delivery systems.

## 2. Results and Discussion

This section presents the results obtained from this study and discusses them. In the first section, the verification of the simulation results is provided to demonstrate the validity of the algorithm and interatomic potentials. In the second and third sections, the diffusivity and the impact of nanobubble cavitation on the nanocapsules were investigated as properties of drug carriers and releasing agents, respectively. These sections facilitate understanding of the diffusion behavior of nanocapsules in pure water and help determine their suitability for rapid diffusion or navigation. Moreover, the investigation of the effects of nanobubble cavitation on nanocapsules, which are used as releasing agent, provides in-depth insight into the underlying physics of this phenomenon and will help improve the release process by understanding its behavior at the molecular scale. In the final section, the outlook and perspectives for future explorations are addressed.

### 2.1. Results Verification

To benchmark the simulation results and evaluate the accuracy of the computed diffusion coefficients, pure water, CN/water, and BNN/water systems were simulated under the diffusion algorithm at environment condition (298 K and 1 atm). Then, the *D* of water molecules was calculated for each system and compared with previously reported data [35,36] in Table 1. As shown in this table, the results obtained in the present study are in good agreement with the earlier data, indicating that the employed potential models and algorithm reliably predict the physical properties water. In addition, single nanobubble cavitation was simulated using the mirror-wall algorithm, and the results were in close agreement with previous findings [37], demonstrating that the cavitation protocol functions correctly and can now be applied to nanocapsule/water systems. Thus, both the diffusion and cavitation algorithms are practical for achieving the objectives of the current study.

The difference in the diffusion coefficients of water molecules between pure water and in the presence of CNs or BNNs can be attributed to atomic interactions, specifically carbon–water interactions in CNs and boron–water and nitrogen–water interactions in BNNs. The Lennard-Jones (12/6) parameters differ for CNs and BNNs, thereby influencing the behavior of water molecules at the nanocapsule–water interface. Due to these interfacial effects, water molecules near the nanocapsule walls exhibit different motion, resulting in variations in the diffusion coefficient. As a result, the diffusion coefficients of water molecules were obtained as 2.22 × 10^−9^ m^2^·s^−1^ for pure water, 2.33 × 10^−9^ m^2^·s^−1^ for CN/water, and 2.50 × 10^−9^ m^2^·s^−1^ for BNN/water under ambient conditions.

As evident, the difference between the diffusion coefficient of water molecules in the presence of CNs and that in pure water is 5%, while for BNNs it is 12%. These relatively small effects (maximum 12%) are induced by surface interactions between the nanocapsules and water molecules. Since the system contains only one nanocapsule, the number of water molecules at the nanocapsule surface is much lower than the total number of water molecules in the bulk medium. In this regard, it can be concluded that BNNs and CNs at low concentrations cannot alter the diffusion coefficient of water molecules by more than 12%. If the contribution of surface interactions increases, for example, by adding more nanocapsules or using larger ones, it can be expected that BNNs and CNs may have a greater effect on the water diffusion coefficient. In summary, it can be concluded that the presence of BNNs or CNs does not significantly affect the diffusion coefficient of water molecules. This is not due to weak interactions between water molecules and the surfaces of BNNs or CNs, but rather because the number of water molecules at the nanocapsule surfaces is overwhelmingly lower than that in the bulk water.

### 2.2. Nanocapsules Diffusivity as Drug Carrier Properties

#### 2.2.1. Carbon Nanocapsules/Water System

**Figure 1:**
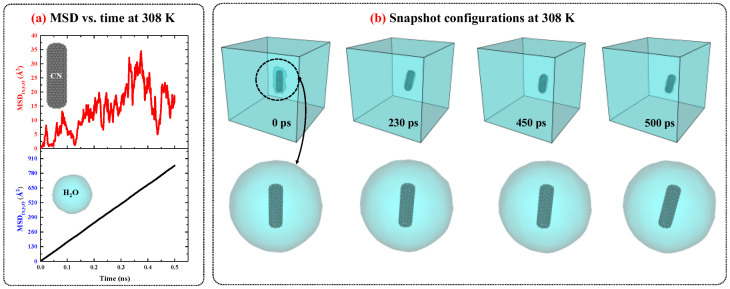
(**a**) The MSD vs. time diagrams for CNs and water in CN/water system at 308 K and 1 atm. (**b**) The snapshots configuration of CN/water system at 308 K and 1 atm as function of time.

Figure 1 presents the MSD versus time diagrams for CNs and water in the CN/water system separately, and illustrates the snapshot configurations of this system at 308 K as a function of time. As shown in Figure 1a, the MSD of H_2_O molecules increases linearly over time with a constant slope, indicating normal diffusion behavior. In contrast, for CN, the increase in MSD over time also suggests diffusion; however, the fluctuations are more pronounced compared to water. A more detailed analysis of the MSD diagrams suggests that these fluctuations may be attributed to changes in the orientation of the CN axis as well as the low concentration of CNs in water.

To provide further evidence regarding this issue, the configuration snapshots over time were captured and plotted in Figure 1b. Based on this figure, the changes and rotational movements around the axis of the CNs are observed over time and influence the MSD. Since, in MSD analysis, particle diffusion is the primary focus, and for water molecules, the rotational movements are smaller than their translational degrees of freedom, the significant contribution to diffusion is attributed to translational motion, resulting in MSD curves with lower thermal fluctuations. On the other hand, for CN, due to its low concentration in water, with only one CN present, the rotational degrees of freedom around its axis affect translational motion and induce fluctuations in the MSD curve. The diffusion behavior of the CN/water system at other temperatures is similar to that observed at 308 K; however, as temperature decreases, both the MSD values and the slopes of their curves decrease accordingly.

**Figure 2:**
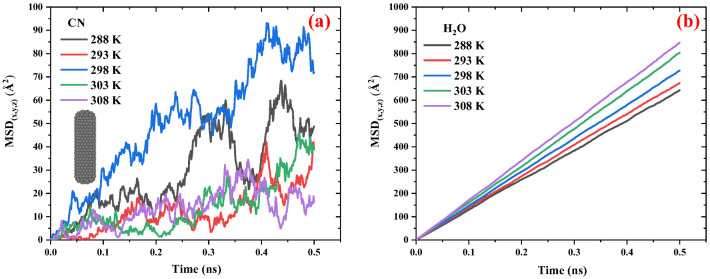
The MSD vs. time diagrams for the (**a**) CNs and (**b**) water in the CN/water system at different temperatures ranging from 298 to 308 K in 1 atm pressure.

Figure 2 compares the MSD vs. time diagrams for CNs and water in the CN/water system at different temperatures ranging from 288 to 308 K. Figure 2a illustrates that, with increasing temperature, the distance between the peaks and valleys of the MSD curves for CN decreases, resulting in a smoother trend. This behavior is attributed to the influence of temperature on CN diffusivity; as temperature increases, translational motion becomes the dominant mode of movement compared to rotational degrees of freedom, thereby reducing large fluctuations in the MSD curves. Although temperature does not exhibit a strictly linear effect on the slope of the MSD diagrams, a slight increase in slope is observed as the temperature rises from 288 K to 298 K. However, in the range from 298 K to 308 K, the slope remains relatively unchanged, indicating a nearly constant diffusion coefficient in this temperature interval. Figure 2b shows that the MSD of H_2_O molecules in the CN/water system follows a more consistent and ordered pattern. With increasing temperature, the slope of the MSD curves increases, indicating enhanced diffusion of water molecules.

To evaluate the effect of temperature on the diffusion coefficients of CN and H_2_O molecules in the CN/water system, the diffusion coefficients were calculated using Equations (1) and (2) and Figure 2 and were listed in Table 2. This table compares the diffusion coefficients of CNs and water in the CN/water system at various temperatures ranging from 298 to 308 K at a pressure of 1 atm. The data show that with a temperature increase of approximately 3.3%, the average diffusion coefficient of CNs is around 0.1 × 10^−9^ m^2^·s^−1^. In contrast, under the same temperature increase, the diffusion coefficient of H_2_O increases from 2.16 to 2.83 × 10^−9^ m^2^·s^−1^ (an approximate rise of 31%). These results indicate that temperature has a significantly greater effect on water molecules than on CNs. Consequently, temperature alone cannot be effectively used as a navigational mechanism for directing CN delivery to cancerous tumors.

#### 2.2.2. Boron-Nitride Nanocapsule/Water System

**Figure 3:**
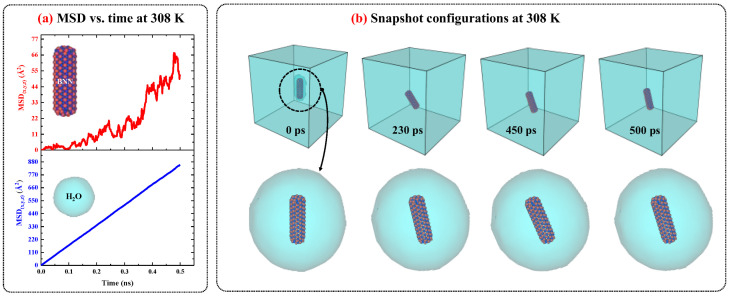
(**a**) The MSD vs. time diagrams for BNNT and water in BNN/water system at 308 K and 1 atm. (**b**) The snapshots configuration of BNN/water system at 308 K and 1 atm as function of time.

Figure 3 illustrates the MSD vs. time diagrams for BNNs and water in the BNN/water system, separately, and shows the snapshot configurations of this system at 308 K as a function of time. The MSD behavior of BNNs and water in the BNN/water system, along with the snapshot configuration analysis, reveals that BNNs exhibit a similar diffusion pattern to CNs when immersed in water. However, BNNs show lower fluctuations and greater diffusion, as indicated by the steeper slope of the MSD vs. time curve for BNNs compared to CNs at the same temperature in Figure 3. The reduced thermal fluctuations of BNNs, in comparison to CNs, can be attributed to their lower rotational motion. Therefore, as rotational movements contribute less significantly, the translational movements dominate, resulting in increased diffusion.

**Figure 4:**
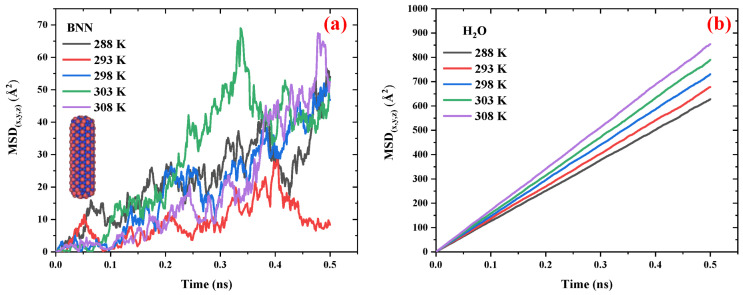
The MSD vs. time diagrams for the (**a**) BNNs and (**b**) water in the BNN/water system at different temperatures ranging from 298 to 308 K in 1 atm pressure.

Figure 4 compares the MSD vs. time diagrams for BNNs and water in the BNN/water system at different temperatures ranging from 298 to 308 K. Figure 4a,b present the MSD of BNN and H_2_O molecules, respectively. A comparison of this figure with Figure 2 shows that the behavior of the BNN/water system is similar to that of the CN/water system; however, BNNs exhibit greater mobility than CNs. In the case of water, the H_2_O molecules display a similar diffusion pattern in the presence of both BNNs and CNs, suggesting that these nanocarriers have no significant effect on their surrounding medium. This outcome is advantageous for drug delivery applications, as it indicates that CNs and BNNs can be introduced into biological environments (composed primarily of water) without significantly disrupting the medium, while still serving effectively as drug carriers.

Table 3 compares the diffusion coefficients of BNNs and water in the BNN/water system at various temperatures, ranging from 298 to 308 K, under a pressure of 1 atm. The results show that when the temperature increases by 3.3%, the diffusion coefficient of H_2_O increases by 31%, while the diffusion coefficient of BNNs remains unchanged. The diffusion coefficient of BNNs across the temperature range of 298–308 K remains constant at 0.11 × 10^−9^ m^2^·s^−1^ m^2^/s. This outcome suggests that temperature has no significant effect on the diffusivity of BNN, presenting both advantages and limitations for drug delivery systems. The advantage is that BNN, as a nanocarrier, can reach cancerous tumors regardless of local temperature fluctuations near tumor sites. Conversely, the limitation is that temperature cannot be used as a navigational stimulus to guide BNNs toward specific targets, reducing the feasibility of thermally driven targeting strategies for this nanocarrier.

Table 3 shows that to evaluate diffusion as a function of temperature, the range of 288–308 K was chosen, as these values are close to the operating temperature range of enzymes in the human body [38,39]. The difference between the minimum and maximum temperature is 20 K, which is insufficient to generate significant thermal energy that could affect diffusion. In simple terms, thermal energy is described by the equation $k_{B}T$, where $k_{B}=8.617333262\times 10^{-5}\frac{eV}{K}$ is the Boltzmann constant and T is the temperature in Kelvin. Based on this relationship, the difference in thermal energy between the minimum and maximum temperatures is $k_{B}\left(308-288\right)K=1.723\times 10^{-3}eV$, which is too small to cause a noticeable effect on diffusion. In this regard, it can be concluded that the diffusion coefficients of BNNs and CNs are effectively temperature-independent in the range of 288–308 K.

#### 2.2.3. Comparison of the Diffusivity of Carbon and Boron-Nitride Nanocapsules

**Figure 5:**
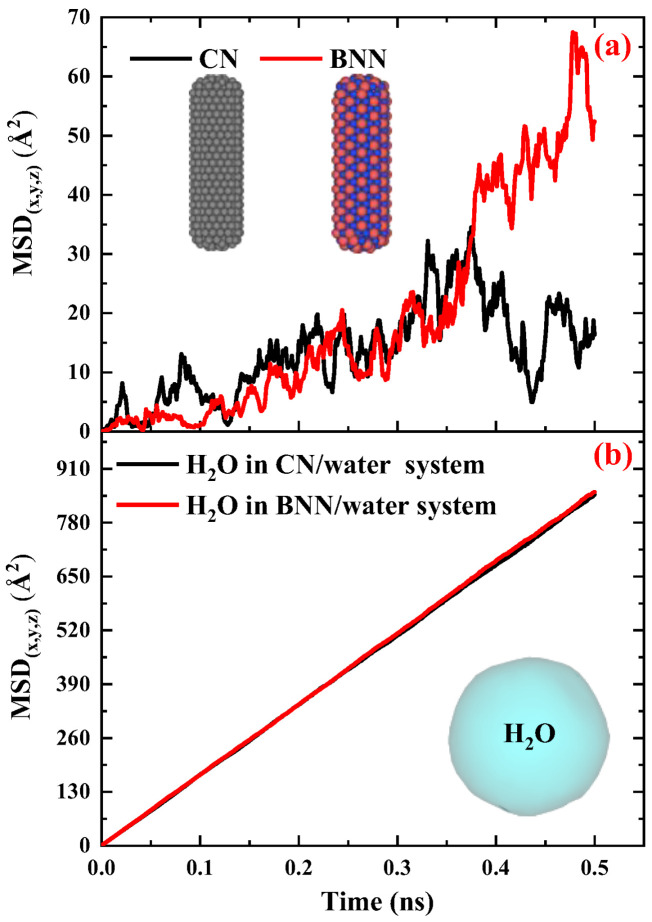
Intercomparison compares the MSD vs. time diagrams for (**a**) CNs and BNNs, along with their (**b**) surrounding water molecules, at 308 K and 1 atm pressure.

Figure 5 compares the MSD vs. time diagrams for CNs and BNN, along with their surrounding water molecules, at 308 K and 1 atm pressure, to provide a clear comparison between these drug nanocarriers and their effects on water. Figure 5a reveals that the MSD of BNNs increases gradually over time, while that of CNs becomes nearly horizontal after 400 ps. This change in MSD indicates that BNNs (0.11 × 10^−9^ m^2^·s^−1^ m^2^/s) have higher mobility compared to CNs (0.10 × 10^−9^ m^2^·s^−1^ m^2^/s), resulting in a greater diffusion coefficient. This behavior can be attributed to the atomic interactions at the nanocapsule–water interface. In the CN–water interface, only carbon atoms interact with water, producing uniform repulsive and attractive forces. However, at the BNN–water interface, both boron and nitrogen atoms interact independently with water, generating a combination of distinct attractive and repulsive forces that enhance mobility and diffusivity. Further investigation into this phenomenon requires bond-order simulations, typically referred to as reactive simulations, which are discussed in the next section. In the case of H_2_O (Figure 5b), the MSD over time in the presence of both BNNs and CNs exhibits an almost identical linear trend, indicating equivalent diffusion coefficients. These results demonstrate that neither BNNs nor CNs have a significant effect on the surrounding water.

In the systems, there are 49,458 water molecules, 1 CN, and 1 BNN. In the presence of CNs and BNNs, the diffusion coefficient of water molecules changes by 5% and 12%, respectively. It should be noted that this difference arises from the collective behavior of all 49,458 water molecules. For a single nanocarrier, BNNs provide a diffusion coefficient approximately 10% higher than that of CNs. This increase for a single nanocarrier is acceptable and noteworthy. The lower fluctuation and rotation observed in BNNs are due to the sliding of water molecules on their surface, which reduces the Brownian effect. In contrast, water molecules in close proximity to CNs experience stronger interactions, inducing greater Brownian motion and resulting in higher fluctuation and rotation.

It is worth noting that, in drug delivery applications, more than one nanocapsule is typically injected in vivo, and the collective behavior of these nanocapsules can be observed, including their interactions and the formation of clusters. However, these nanocapsules eventually break down individually, releasing the drug. In this regard, a single nanocapsule was simulated to investigate the behavior of an individual nanocarrier. Regarding nanocapsule concentration, an increase in the number of nanocapsules leads to different trends in the MSD diagrams and the diffusion coefficient. When multiple nanocapsules are present, cluster diffusion and collisions can be expected; these factors in turn influence the diffusion coefficient. The collective behavior of nanocapsules differs from that of a single nanocapsule, and this represents a promising opportunity for future research in this field.

### 2.3. Nanobubble Cavitation as Releasing Agent

**Figure 6:**
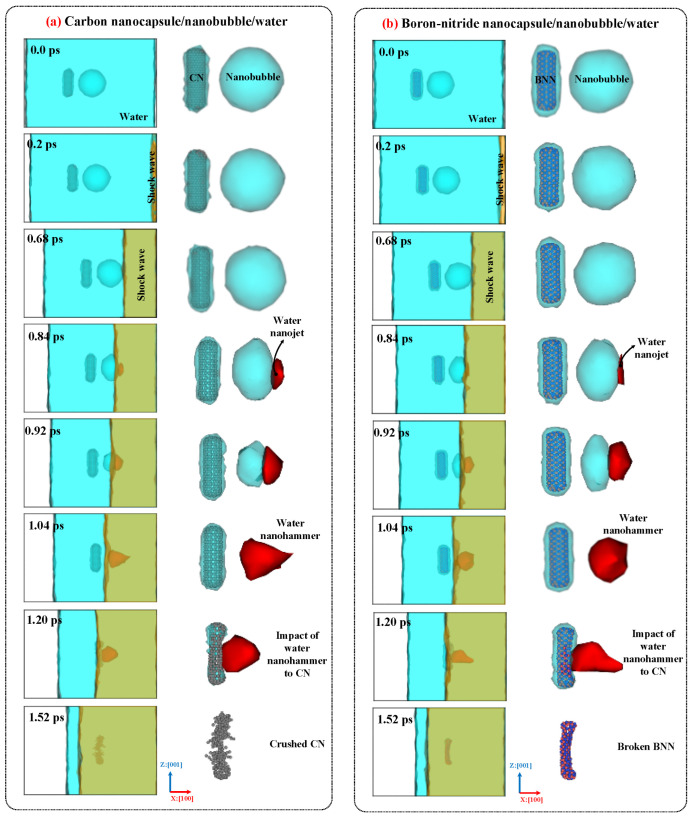
The dynamics, collapse mechanism, formation of the water nanojet, impact of the water nanohammer, and damage mechanism for (**a**) CN/nanobubble/water and (**b**) BNN/nanobubble/water systems at 298 K and 1 atm pressure.

Figure 6 illustrates the collapse dynamics, formation of the water nanojet, emergence of the water nanohammer, collision of the nanohammer with the nanocapsules, and nanocapsule-induced damage caused by the nanohammer during nanobubble cavitation at 298 K and 1 atm. As shown in the figure, the movement of the entire system toward the mirror wall along the x:[100] direction leads to the collision of water molecules with mirror wall. As a result, the momentum of the water molecules is altered upon collision, and a planar shock wave forms and propagates along the x:[$\bar{1}$00] direction. The orange mesh color in the figure illustrates this shock wave. Then, the shock wave moves upward and eventually reaches the upper wall of the nanobubble, where it collides with the nanobubble–water interface. Upon this impact, the nanobubble begins to collapse, and its volume decreases as the pressure exerted by the shock wave exceeds the internal pressure of the nanobubble’s wall.

As the nanobubble volume decreases (as evident in Figure 6 between 0.68 and 1.04 ps), a water nanojet forms (highlighted by the red mesh in the figure) on the side where the nanobubble begins to collapse. This occurs because water molecules move rapidly into the vacated volume of the nanobubble, encountering little resistance, which leads to an increase in their velocity and the formation of a focused jet. The volume of the water nanojet gradually increases as the collapse proceeds. When the nanobubble entirely collapses at 1.04 ps, the nanojet reaches its maximum volume and is typically referred to as the “water nanohammer”, as it is responsible for the final impact and damage. Ultimately, the water nanohammer strikes the nanocapsules, breaking them and thereby creating favorable conditions for the release of the drug.

Comparing the time-evolved snapshots for the CN/nanobubble/water and BNN/nanobubble/water systems in Figure 6 reveals that the nanobubble cavitation behavior in both cases is similar, and the type of nanocapsule material (CN or BNN) does not significantly affect the collapse dynamics. However, the damage induced by the water nanohammer differs markedly between the two systems. At the end of the nanobubble collapse, the water nanohammer forms and moves toward both CNs and BNNs with approximately equal volume and energy; nevertheless, it interacts with them differently. As shown in this figure, when the water nanohammer transfers all its energy to the nanocapsules at the final stage (1.52 ps), the CN nanocapsule is crushed, whereas the BNN nanocapsule is broken.

This outcome suggests that the water nanohammer collides with greater intensity with CNs compared to BNNs. The origin of this phenomenon can be attributed to the nature of atomic interactions between the water nanohammer and the constituent atoms of the nanocapsules. Carbon atoms in CNs interact uniformly with water molecules at the leading edge of the nanohammer, whereas boron and nitrogen atoms in BNNs exhibit different interaction behaviors. As a result, the momentum transfer from the water nanohammer is more focused in the CN system and more dispersed in the BNN system. Consequently, the CN is crushed, while the BNN is merely broken. Crushing was defined as the splitting of a sample into more than five pieces, accompanied by atomic branching and the release of free atoms after collapse. Breakage was defined as the splitting of a sample into two pieces without atomic branching or the release of free atoms.

The crushing of CNs indicates that this system can facilitate efficient drug release under cavitation conditions. However, such crushing may also pose a risk of damaging the encapsulated anticancer drug. Before the impact of the water nanohammer on CN, the maximum internal distance between the walls of CNs (the diameter) is 0.8 nm. However, after the impact of the water nanohammer, the internal distance between the walls decreases to below the van der Waals (vdW) distance between graphene layers (0.34 nm) [40], leaving no space for atomic species to remain. As a result, the compression is sufficiently high to bring carbon atoms closer together than the vdW length. In this context, if encapsulated medications were present between the crushed walls, they would experience high pressure, and potential damage could be expected. It is worth noting that this conclusion is based on vdW analysis. To provide additional criteria, simulations need to be repeated with encapsulated medications to observe the outcomes directly. In contrast, the structural failure in BNN, characterized by wall breakage rather than crushing, may enable drug release with reduced risk of molecular damage.

Figure 6 illustrates that the boundary water layer around CN and BNN differs at the instant of nanohammer impact. To investigate the reason for these differences in boundary water distribution, radial distribution function (RDF) [41] analysis was performed. This analysis shows that the minimum distance between CNs and water in the CN/nanobubble/water system is 26% lower than the corresponding distance between BNNs and water in the BNN/nanobubble/water system. This result demonstrates that water molecules are closer to CNs compared to BNNs, and, as a consequence, the impact of the momentum is more effectively transferred over this shorter distance. Because the distance between C atoms and water molecules in the CN/nanobubble/water system is approximately equal to the vdW distance (0.247 nm), the momentum of the water molecules in the water nanohammer is directly transferred to the carbon atoms in CNs. As a result, CNs absorb a large portion of the nanohammer’s energy, leading to effective crushing. However, in the BNN/nanobubble/water system, the distance of water molecules from B and N atoms (0.39 nm) exceeds the average vdW distance (0.31 nm). This creates a relatively thin and flexible interfacial region that allows the water molecules from the nanohammer to slide, dispersing a portion of their momentum due to the differing attractive and repulsive interactions induced by the B and N atoms. Consequently, the impact is reduced, and BNN undergoes breakage rather than crushing.

**Figure 7:**
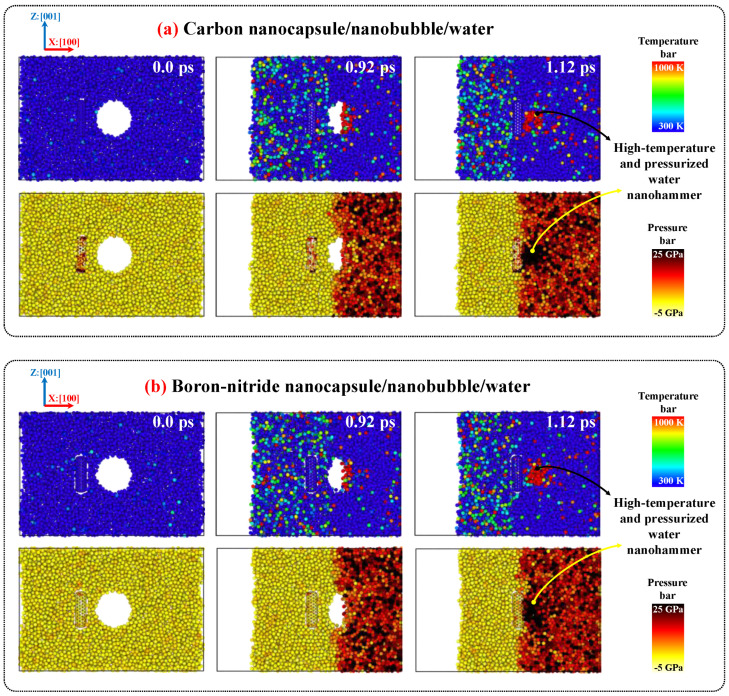
The temperature and pressure maps for (**a**) CN/nanobubble/water and (**b**) BNN/nanobubble/water systems at 298 K and 1 atm pressure during nanobubble cavitation.

To provide further evidence regarding the causes of crushing and breakage in CN and BNN, respectively, Figure 7 compares the temperature and pressure profiles of the CN/nanobubble/water and BNN/nanobubble/water systems during cavitation. As shown in the figure, the pressurized shock wave initiates the collapse of the nanobubble. Once the collapse begins, the resulting water nanojet displays high pressures and elevated temperatures, reaching up to 25 GPa and 1000 K, respectively. At the end of the collapse, this high-energy nanojet transforms into the water nanohammer, which transfers its thermal energy and pressure to the nanocapsules upon impact, causing structural damage. In the CN system, due to the localized nature of the collision, the nanocapsule absorbs nearly all of the transferred energy and pressure, resulting in complete crushing. In contrast, the BNN system disperses a portion of the temperature and pressure upon impact, leading to breakage rather than total collapse.

The results obtained in the present study can be qualitatively extrapolated to larger capsule sizes; however, quantitative data cannot be directly extrapolated. The findings indicate that BNNs exhibit better diffusion characteristics and provide more favorable conditions for drug release under nanobubble cavitation compared to CNs. This outcome arises from the inherent properties of BNNs and CNs, as their interactions with water and cavitation nanobubbles are consistent, allowing qualitative results to be extended to larger capsules. However, diffusion coefficients and the intensity of damage induced by nanobubble cavitation vary with capsule size. As the capsule size increases, more energy is required to induce diffusion, and a stronger water nanohammer is necessary to generate damage. This is because a greater amount of material, with more linking bonds, can withstand higher mechanical and thermal stresses compared to smaller capsules. Therefore, it can be concluded that the general qualitative findings, namely, the better diffusion characteristics and favorable conditions for drug release exhibited by BNNs compared to CNs, can be extrapolated to larger nanocapsules.

### 2.4. Outlook and Perspective

**Figure 8:**
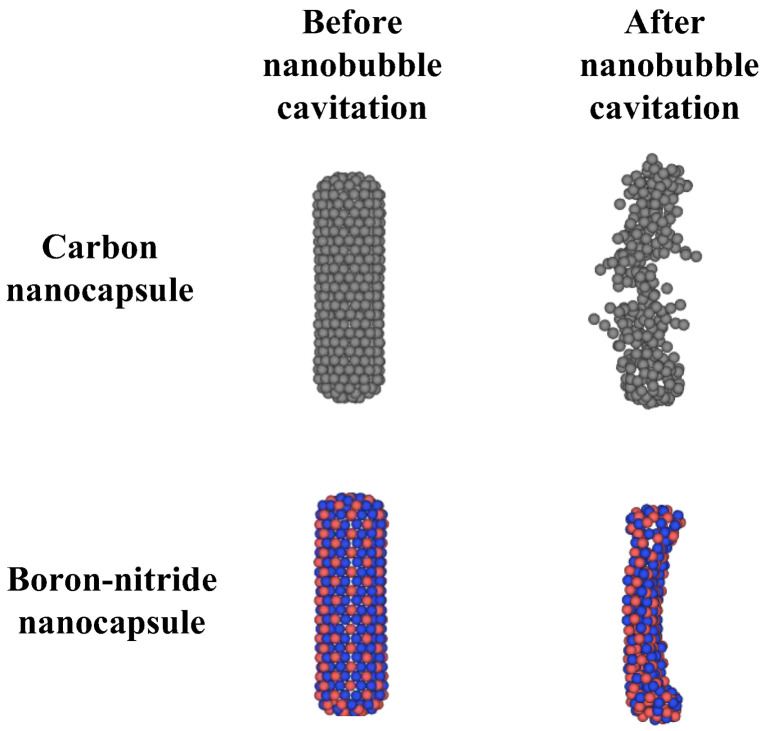
Comparison of carbon (BN) and boron-nitride (BNN) nanocapsules before and after cavitation damage by nanobubble collapse under a shock wave with a velocity of 3 nm/ps.

The main findings indicate that BNNs diffuse more than CNs, and that neither nanocapsule significantly influences its surrounding medium. Additionally, temperature cannot be utilized as a navigation parameter for either system. Under nanobubble cavitation, CNs undergo crushing, while BNNs experience breakage. Figure 8 compares the configurations of CNs and BNNs before and after cavitation-induced damage. The crushing and breaking of CNs and BNNs, respectively, provide favorable conditions for the release of the drug. However, the complete crushing of CNs increases the possibility of damage to the encapsulated drug. It is now necessary to develop a strategy for navigating CN and BNNs and to further investigate the drug release mechanisms from these nanocapsules, as well as the potential for drug damage under nanobubble cavitation.

In the case of actual biological nanocarriers, the choice of chiral indices and nanocapsule dimensions is critical and must be made in relation to the type and size of the drug. To address navigation challenges, functional groups can be added to CNs or BNNs to activate them for chemical reactions and to render them responsive to external electric or magnetic fields. When functional groups containing metallic atoms such as iron or nickel are introduced [42,43,44], both of which respond to external electromagnetic fields, they enable the nanocapsules to alter their direction and move along a desired pathway under the influence of these fields. This mechanism facilitates controlled navigation and targeted delivery to cancerous tumors, thereby enhancing the overall performance of drug delivery.

To minimize drug degradation caused by collapse-induced shock, alternative release mechanisms, such as neutron radiation [45,46,47], may be employed. This approach can be efficient for BNNs, due to the presence of B, which is capable of absorbing neutron radiation. Upon interaction with neutron-rays, B atoms can facilitate localized wall breakage, triggering drug release without damaging the drug itself, especially since the drug molecules do not contain B and therefore do not absorb neutron radiation. Furthermore, this method requires reactive MD simulations to confirm whether new chemical species are formed following wall breakage and drug release. These proposed strategies are promising for future applications in drug delivery systems utilizing CNs and BNNs.

In the case of materials, it can be expressed that various materials can serve as nanocarriers such as: peptides [48], luminescent and mesoporous core–shell structured composites [49], metal–organic frameworks [50], CNs, silicon carbide, and BNNs [4,22,23,25,27]. Among these, CNs [51,52,53,54] and BNNs [55,56,57,58,59] are not only widely used but also capable of enabling drug release through both radiation and cavitation mechanisms. This paper focuses on investigating the diffusion properties of CNs and BNNs, as well as the effects of cavitation, thereby laying the groundwork for further research. In the future stages, the effects of radiation on drug release can be investigated. A comparative analysis of these studies will clarify, for CNs and BNNs, which material exhibits more favorable self-diffusion properties and which drug-release mechanism is more suitable.

This study was conducted in pure water, whereas results should ideally be provided in real biological environments such as serum and interstitial fluid to ensure more accurate insights regarding drug release. However, within the scope of this study, as one of the pioneering efforts to investigate the effects of cavitation on CNs and BNNs, pure water was selected because the human body is primarily composed of water. Although the general mechanism of cavitation is similar in pure water, serum, and interstitial fluid, the specific conditions and effects differ due to variations in density and impurities within these systems. Consequently, while nanobubbles can form and induce damage on CNs and BNNs in all these environments, the extent and nature of the damage may vary. To address this issue more comprehensively, further simulations under more realistic conditions, specifically in serum and interstitial fluid, are required to better capture the interactions between nanobubble cavitation and drug nanocarriers in biological environments.

It should be noted that, from the injection site to the tumor area, the drug travels through in vivo environments that typically exhibit temperatures within the range of 288–308 K, as these values are close to the operating temperature range of enzymes in the human body [38,39]. The results obtained within this range provide insights into the behavior of the nanocarriers during the transport stage in vivo. Moreover, to present a general overview of the release phase, ambient conditions were used as a standard to describe the interactions between nanocapsules and water nanohammers induced by nanobubble collapse. However, the release phase must occur under tumor microenvironment conditions, which vary depending on the tumor type. In this regard, it can be inferred that reproducing the simulations under tumor microenvironment conditions could yield more accurate results for both the drug transport and release stages.

## 3. Materials and Methods

### 3.1. Configuration

To model the water/nanocapsule configurations, water was defined using a coarse-grained (CG) approach [60]. In contrast, the carbon nanocapsule (CN) and boron-nitride nanocapsule (BNN) were modeled in an all-atom approach, with chiral indices of (n = 6, m = 6), diameters of 0.8 nanometers (nm), and lengths of 2.9 nm. Using similar nanocapsules allows for a comparable evaluation of the diffusion properties of both, helping to determine which is more suitable for drug carrier applications. The size of the simulation box for the diffusion study was set to 10 × 10 × 10 nm^3^, which is sufficiently large to maintain the bulk behavior of pure water [35]. The nanocapsules (CN or BNN) were placed at the center of the simulation box, and the remaining space was filled with water molecules at a density of 0.997 g/cm^3^ [37,41] in two independent simulations (one for CN/water and the other for BNN/water).

**Figure 9:**
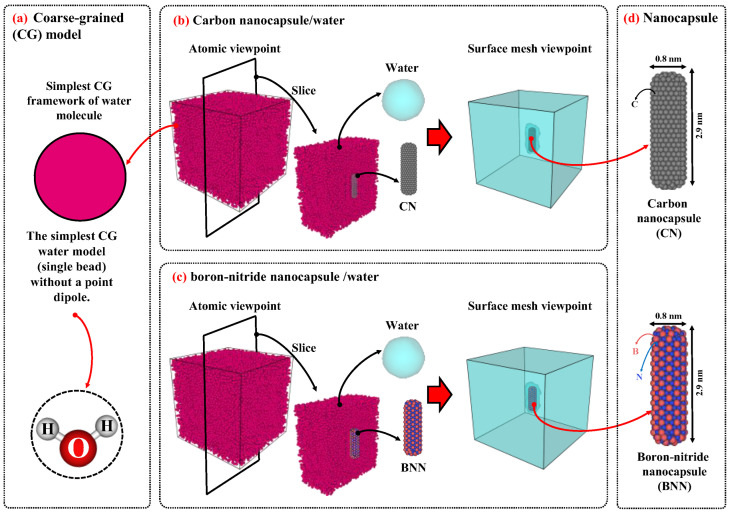
(**a**) The schematic of coarse-grained (CG) model. The initial configurations of (**b**) carbon nanocapsule (CN)/water and (**c**) boron-nitride nanocapsule (BNN)/water. (**d**) Carbon and boron-nitride nanocapsules were modeled in an all-atom approach, with chiral indices of (n = 6, m = 6), diameters of 0.8 nanometers (nm), and lengths of 2.9 nm. The size of the simulation was set to 10 × 10 × 10 nm^3^ and filled with water CG bead corresponding to 0.997 g/cm^3^.

**Figure 10:**
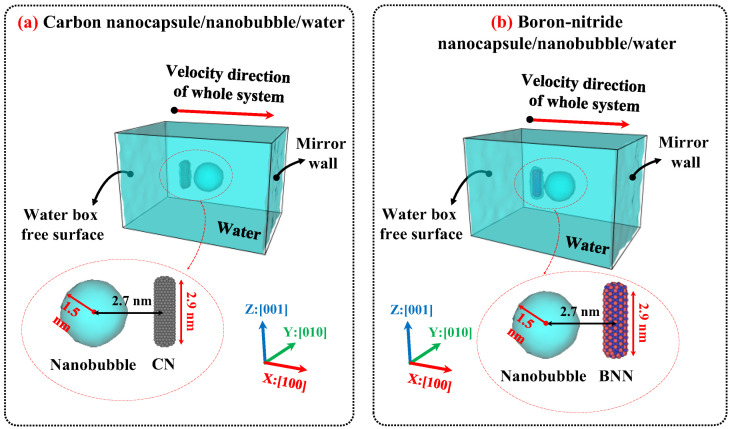
Initial configuration of (**a**) carbon nanocapsule (CN)/nanobubble/water and (**b**) boron-nitride nanocapsules (BNN)/nanobubble/water after creating nanobubble. Carbon and boron-nitride nanocapsules were modeled in an all-atom approach, with chiral indices of (n = 6, m = 6), diameters of 0.8 nanometers (nm), and lengths of 2.9 nm. The size of the simulation was set to 10 × 10 × 15 nm^3^ and filled with water CG bead corresponding to 0.997 g/cm^3^. The distance between the center of the nanobubble and the axis of the nanocapsules was set to 2.7 nm. The initial radius of the nanobubble is 1.5 nm. The gamma distance (the relative wall distance, defined as the distance between the center of the nanobubble and the axis of the nanocapsules (2.7 nm) divided by the radius of the nanobubble (1.5 nm)) is equal to 1.8.

Figure 9 illustrates the initial configurations of CN/water and BNN/water. The configuration for studying bubble cavitation effects is similar to that used in the diffusion simulations; however, the simulation box was set to 10 × 10 × 15 nm^3^ to provide sufficient space for shock wave propagation and interaction with the nanocapsules. Figure 10 illustrates the initial configurations used for nanobubble cavitation studies. As a foundation for the current simulations, nanocapsules with a length of 2.9 nm and nanobubbles with a diameter of 3.0 nm were selected. Accordingly, the distance between the center of the nanocapsule and the center of the nanobubble was set to approximately 2.7 nm. As a result, the system was modeled with consistent nanocapsule length, nanobubble radius, and wall distance between the nanocapsule and the nanobubble. It is worth noting that variations in any of these parameters can influence the simulation outcomes.

### 3.2. Interatomic Potential

To model the interaction between carbon (C) atoms in CN, the adaptive intermolecular reactive empirical bond order (AIREBO) potential was used [61,62]. This potential can accurately predict the physical properties of low-dimensional carbon materials. For modeling the interaction between boron (B) atoms and nitrogen (N) atoms, as well as B–N interactions in BNN, the extended bond-order Tersoff potential [63] was employed due to its accuracy in modeling interactions within this system and its ability to account for the formation and breaking of B–N bonds [64]. One of the key potential functions fully incorporated in the AIREBO and Tersoff potentials is the bonding potential and its associated energies. These potentials can intelligently identify C–C, B–B, B–N, and N–N distances and estimate bond states with respect to the geometric configuration at each timestep when the potential is applied and the Newtonian equations of motion are solved. If the geometry is suitable and the atoms are close enough to form bonds, the potential accounts for the corresponding bond energy. Conversely, if the distance between two atoms exceeds the standard values defined in the potentials, the bond energy is not considered. Therefore, the bond energies constituting CNs and BNNs are fully accounted for in the simulations, both in the diffusion and cavitation analyses.

The interaction between water molecules was also modeled using the CG approach proposed by Zavadlav et al. [60]. This potential can reproduce the thermodynamic properties of water closely to all-atom models while significantly reducing computational costs, making it useful for simulating large systems [41]. The interaction between water CG beads and C, B, and N atoms was defined by non-bonding Lennard-Jones “12/6” potentials using the Lorentz–Berthelot mixing rules [35] to model water–solid interphase interactions [60,65].

### 3.3. Simulation Algorithm

All molecular dynamics (MD) simulations were performed using the LAMMPS package (Version 19Nov2024-MSMPI) [66], and OVITO (Version 3.0.0-dev234) [67] software was used for visualization. Two separate algorithms were employed for diffusion and cavitation purposes. In the diffusion algorithm, the minimization, equilibrium, and diffusion stages were defined. In contrast, the cavitation algorithm replaced the final stage with a shock wave propagation stage. For computing diffusion properties, periodic boundary conditions (PBCs) were applied in all directions to ensure that the bulk behavior of water remained consistent near the boundaries. Prior to the main simulation, a combination of the microcanonical (NVE) ensemble and the Langevin thermostat was used to reduce the system’s energy from a metastable to a stable microstate over 1 picosecond (ps) with a time step of 0.1 femtoseconds (fs) [37]. After achieving the stable microstate, the NVE and Langevin thermostat were turned off, and the time step was changed to 1 fs. Subsequently, the isothermal–isobaric ensemble (NPT) with the desired temperature and a pressure of 1 atmosphere was applied to the system for 2 nanoseconds (ns) to achieve thermodynamic equilibrium. This time is sufficient to ensure the system reaches equilibrium. Finally, the NPT was turned off, and the NVE was used for 1 ns to calculate the diffusion coefficient. To evaluate diffusion as a function of temperature, the range of 288, 293, 298, 303, and 308 K was chosen, as these values are close to the operating temperature range of enzymes in the human body [38,39].

For nanobubble cavitation, the minimization and equilibration stages were performed as in the diffusion algorithm. However, after reaching thermodynamic equilibrium, a mirror-wall protocol was applied to generate a shock wave [37]. The simulations for nanobubble cavitation were performed at 298 K and 1 atm. Periodic boundary conditions along x:[100] were replaced with fixed boundaries, and two virtual mirror walls were defined at the lower and upper faces of the simulation box in this direction. A spherical region (radius = 0.8 nm) was created near the nanocapsules, and all water molecules within it were removed, producing a void that served as the nanobubble. Using this method, the void nanobubble with a gamma distance (relative wall distance) of 1.8 was created. The vapor phase inside the bubble was not explicitly modeled because its density is extremely low; including it would slightly reduce the post-collapse jet pressure but would not change the qualitative cavitation behavior [68]. The entire system was then given an initial center-of-mass velocity of 3 nm/ps (3 km/s) along the x:[100] axis toward the upper mirror wall. Upon impact, the water molecules reflected, reversing their momentum and generating a shock wave that propagated in the opposite direction along x:[$\bar{1}$00]. When the shock front reached the nanobubble interface, the bubble collapsed, forming a water nanojet that impacted the nanocapsules.

### 3.4. Formal Analysis

In this study, two classes of formal analysis were utilized to investigate the diffusion behavior and the effects of cavitation. For the diffusion analysis, the mean square displacement (MSD), which reveals the displacement and diffusion of each particle (water CG bead, CN, and BNN), was computed during the diffusion stage and then plotted against simulation time to determine the diffusion coefficient. Equation (1) illustrates the MSD equation used in the molecular dynamics (MD) simulation [40,69].

(1) $$MSD\left(t\right)=\frac{1}{N}\sum_{i=1}^{N}\left[{\vec{r}}_{i}\left(t+t_{0}\right)-{\vec{r}}_{i}\left(t_{0}\right)\right]$$

Here, N is the number of particles for which the MSD is computed, ${\vec{r}}_{i}$ denotes the position vector of the *i*th particle, t signifies the time, and $t_{0}$ points to the initial time. The slope of the MSD versus time can be used to determine the diffusion coefficient based on the following equation:

(2) $$MSD\left(t\right)=2nDt+\beta$$

where *MSD* is a function of time, n signifies the dimension, *D* is the diffusion coefficient, and β is the thermodynamically sensitive factor that depends on temperature, density, impurities, or other environmental variables. For the cavitation part, the dynamics and phenomena were evaluated using visualization snapshots, velocity maps, pressure contours, and temperature profiles. Each of these formal analyses helps in revealing the cavitation dynamics and thermodynamic criteria during collapse, which facilitates the study of the interaction between nanobubbles and nanocapsules. More information regarding the post-processing analysis methods can be found elsewhere for shock wave and laser-induced cavitation methods [37,41].

## 4. Conclusions

The current study investigates the diffusion behavior of CNs and BNNs as drug carriers, as well as the effects of cavitation nanobubbles on them, to provide in-depth theoretical insight into drug delivery and release strategies using MD simulations. The investigation of the diffusion behavior of the CN/water system over the temperature range of 298–308 K reveals that CN nanocarriers exhibit both translational and rotational degrees of freedom; as temperature increases, translational motion becomes dominant. A 3.3% rise in temperature increases the diffusion coefficient of water by 31% (from 2.16 × 10^−9^ to 2.83 × 10^−9^ m^2^·s^−1^), while having no significant influence on CNs (0.1 × 10^−9^ m^2^·s^−1^), indicating that temperature has a more substantial effect on water than on CNs.

The examination of the diffusion characteristics of the BNN/water system reveals that, in all cases, BNNs exhibit similar behavior to CNs; however, their diffusion coefficient (D_BNN_ = 0.1 × 10^−9^ m^2^·s^−1^) is approximately 1% higher than that of CNs and remains independent of temperature. The differing diffusivity behaviors of CNs and BNNs can be attributed to variations in interaction forces at the CN–water and BNN–water interfaces, respectively. In the CN–water system, only carbon atoms interact with water, whereas in the BNN–water system, both boron and nitrogen atoms participate in interactions with water.

H_2_O also displays nearly identical behavior and diffusion coefficients in the presence of both BNNs and CNs. These results can be categorized into two types: positive and negative. Regarding the adverse outcomes, the findings suggest that temperature variations, such as those occurring in tissues near cancerous tumors, are unlikely to facilitate the targeted delivery of CN or BNN nanocarriers; thus, temperature alone is not an effective navigational cue for these drug delivery systems. On the positive side, these drug nanocarriers have no significant influence on their medium and can be used in drug delivery systems without altering the behavior of their surrounding environment.

The investigation of the effects of nanobubble cavitation on CNs and BNNs as drug-releasing agents at 298 K and 1 atm reveals that these nanocapsules do not influence the dynamics of nanobubble behavior or collapse. However, the water nanohammer, formed following the collapse of nanobubbles and moving toward the nanocapsules at a temperature of approximately 1000 K and a pressure of 25 GPa, induces different types of damage. Under the impact of the water nanohammer, CNs are crushed, while BNNs undergo breakage. This difference arises from the distinct interaction mechanisms at the nanohammer interface: C–water interactions in the CN/nanohammer system differ fundamentally from B–water and N–water interactions in the BNN/nanohammer system. The crushing of CNs under cavitation facilitates efficient drug release; however, it may also increase the risk of damage to the encapsulated anticancer drug. In contrast, the wall breakage observed in BNNs provides favorable conditions for drug release while potentially minimizing the risk of damage to the encapsulated therapeutic agents.

In summary, this study concludes that BNNs exhibit better diffusion characteristics and offer more favorable conditions for drug release under nanobubble cavitation compared to CNs. However, challenges related to navigation, targeted delivery to cancerous tumors, and the safe release of drugs without damaging the encapsulated drug remain to be addressed. To address these challenges, functional groups containing metallic atoms such as iron or nickel may be employed to enable navigation and tumor targeting under external electromagnetic fields. Additionally, neutron radiation may serve as an alternative mechanism for controlled drug release. These aspects require further investigation in future research on drug delivery systems to establish a comprehensive and safe framework for biomedical applications, particularly those intended for use in the human body.

## Figures and Tables

**Table 1:** Intercomparison of the diffusion coefficient of water molecules computed in the current study with previous experimental and computational studies [35,36].

Study	This Work			References		
System	Pure Water	CN/Water	BNN/Water	Pure Water ^a^	Saline Water ^a^	Saline Water ^b^
D_H2O_ (10^−9^ m^2^·s^−1^)	2.22	2.33	2.50	2.29	2.01	2.03

^a^ Experiment: [35,36]. ^b^ Simulation: [35].

**Table 2:** Comparing the diffusion coefficient of CNs and water in the CN/water system at different temperatures ranging from 298 to 308 K at 1 atm pressure.

Temperature (K)	288	293	298	303	308
D_CN_ (10^−9^ m^2^·s^−1^)	0.11	0.08	0.12	0.10	0.09
D_H2O_ (10^−9^ m^2^·s^−1^)	2.16	2.16	2.33	2.66	2.83

**Table 3:** Comparing the diffusion coefficient of BNNs and water in the BNC/water system at different temperatures ranging from 298 to 308 K at 1 atm pressure.

Temperature (K)	288	293	298	303	308
D_BNN_ (10^−9^ m^2^·s^−1^)	0.11	0.10	0.11	0.11	0.11
D_H2O_ (10^−9^ m^2^·s^−1^)	2.16	2.16	2.50	2.66	2.83

## Data Availability

The original contributions presented in this study are included in the article. Further inquiries can be directed to the corresponding author.
